# Insulin inhibits inflammation-induced cone death in retinal detachment

**DOI:** 10.1186/s12974-020-02039-1

**Published:** 2020-11-26

**Authors:** Jean-Baptiste Conart, Guillaume Blot, Sébastien Augustin, Géraldine Millet-Puel, Christophe Roubeix, Fanny Beguier, Hugo Charles-Messance, Sara Touhami, José-Alain Sahel, Jean-Paul Berrod, Thierry Léveillard, Xavier Guillonneau, Cécile Delarasse, Florian Sennlaub

**Affiliations:** 1 Institut de la Vision, INSERM, UMR_S 968, CNRS, Sorbonne Université, 17 rue Moreau, F-75012 Paris, France; 2 Département d’Ophtalmologie, CHRU Nancy, Allée du Morvan, Vandoeuvre-lès-Nancy, France

**Keywords:** Retinal detachment, Cone degeneration, Inflammation, Mononuclear phagocytes, Insulin signaling

## Abstract

**Background:**

Rhegmatogenous retinal detachment (RD) involving the macula is a major cause of visual impairment despite high surgical success rate, mainly because of cone death. RD causes the infiltration of activated immune cells, but it is not clear whether and how infiltrating inflammatory cells contribute to cone cell loss.

**Methods:**

Vitreous samples from patients with RD and from control patients with macular hole were analyzed to characterize the inflammatory response to RD. A mouse model of RD and retinal explants culture were then used to explore the mechanisms leading to cone death.

**Results:**

Analysis of vitreous samples confirms that RD induces a marked inflammatory response with increased cytokine and chemokine expression in humans, which is closely mimicked by experimental murine RD. In this model, we corroborate that myeloid cells and T-lymphocytes contribute to cone loss, as the inhibition of their accumulation by Thrombospondin 1 (TSP1) increased cone survival. Using monocyte/retinal co-cultures and TSP1 treatment in RD, we demonstrate that immune cell infiltration downregulates rod-derived cone viability factor (RdCVF), which physiologically regulates glucose uptake in cones. Insulin and the insulin sensitizers rosiglitazone and metformin prevent in part the RD-induced cone loss in vivo, despite the persistence of inflammation

**Conclusion:**

Our results describe a new mechanism by which inflammation induces cone death in RD, likely through cone starvation due to the downregulation of RdCVF that could be reversed by insulin. Therapeutic inhibition of inflammation and stimulation of glucose availability in cones by insulin signaling might prevent RD-associated cone death until the RD can be surgically repaired and improve visual outcome after RD.

**Trial registration:**

ClinicalTrials.govNCT03318588

## Background

Rhegmatogenous retinal detachment (RD) is a sight-threatening condition with an annual incidence of 10.5 per 100,000 people [1]. Advances in surgical techniques over recent decades have greatly improved the anatomical results with a primary success rate currently up to 80% [2]. However, despite a successful retinal reattachment, visual recovery may still be disappointing, especially in cases involving the cone-rich macula [3] and this loss of vision is primarily due to photoreceptor cell death [4, 5]. Several pathogenic mechanisms have been identified but accumulating evidence suggests that inflammation plays a key role in the pathogenesis of RD-induced rod-photoreceptor cell death. Human studies have thus reported elevated levels of cytokines and chemokines in the vitreous of patients with RD [6–9]. Furthermore, experimental models have found a similar increase in cytokine mRNA expression, as well as attraction of leukocytes to the retina and migration of mononuclear phagocytes (MPs) into the subretinal space [7, 10]. It has also been demonstrated that infiltrating MPs and associated cytokines induce some degree of death of rods following retinal detachment [11–14].

Although cones represent only 5% of all photoreceptor cells in humans, they are responsible for daylight, high-acuity, and color vision. In retinal detachment, cone density decreases despite successful surgery and there is a strong correlation between post-operative cone density and visual acuity [15]. It is therefore important to identify the yet unknown mechanisms of RD-associated cone death to establish therapeutic targets for preventing visual impairment.

Cones are highly metabolically active cells which particularly depend on glucose for function and long-term survival [16, 17]. Contrary to muscle and fat (in which glucose absorption is mediated by the insulin-dependent Glucose transporter 4 (GLUT4)), glucose uptake in adult neurons depends mainly on the insulin-independent GLUT3 [18]. Interestingly, cone glucose uptake relies on GLUT1, which is regulated by the Rod-derived cone viability factor (RdCVF) [16, 17] and the Insulin/mTOR pathway [19, 20]. RdCVF is encoded by the nucleoredoxin-like 1 (NXNL1) gene. Its short splice variant RdCVF, which lacks the thioredoxin motive, is secreted by rods and interacts specifically with basigin 1 and GLUT1 (SLC2A) on cones, stimulating the transporter activity of GLUT1 [16]. RdCVFL, the long splice form, which contains the thioredoxin motive, is expressed in rods an cones and protects against oxidative stress [21, 22]. Protection against oxidative stress also helps maintain glucose availability, as oxidative stress (that plays an important role in RD [23]) markedly decreases the half-life of GLUT1 at the plasma membrane and inhibits GLUT1-dependent glucose uptake (shown in vascular endothelial cells) [24]. Additionally, cones have their own endogenous insulin receptor signaling pathway including the phosphoinositide 3-kinase (PI3K) and m-TOR and cone-specific deletion of PI3K (p85) is sufficient to induce age-related cone degeneration [25, 26]. In retinitis pigmentosa, where RdCVF levels are extinguished due to the primary loss of rods, systemic administration of insulin improved glucose uptake in cones and delayed their death, despite the absence of RdCVF [19, 20]. Together, these studies reveal the crucial role of glucose availability in cone viability.

Inflammatory cells are also very reliant on glucose for metabolic activity, in particular when activated [27]. In inflamed tissue, leukocytes can additionally reduce glucose uptake in adjacent stromal cells as their cytokines, such as IL-1β, IL-6, and IFN-γ, inhibit insulin signaling, an important mechanism of type 2 diabetes [28]. In RD, it is not clear whether and how infiltrating inflammatory cells contribute to cone cell loss.

We here demonstrate, using human vitreous samples and a mouse model of RD, that RD causes a severe inflammatory response characterized by increased cytokine expression. In experimental RD, we demonstrate that the recruitment of myeloid cells and T-lymphocytes is closely associated with cone loss and diminished expression of RdCVF and RdCVFL in RD, which are in part restored when the accumulation of inflammatory cells is inhibited by Thrombospondine 1 (TSP1). We confirm that cones are highly dependent on insulin signaling for survival in vitro and demonstrate that insulin and the insulin sensitizers rosiglitazone and metformin prevent RD-induced cone loss in vivo, despite the persistence of retinal inflammation. Taken together, our results strongly suggest that infiltrating immune cells contribute to cone death by reducing the availability of glucose in cones likely through competition and downregulation of RdCVF. Improving glucose uptake by insulin or insulin sensitizers might represent a therapeutic target for delaying RD-associated cone degeneration and subsequent vision loss.

## Methods

### Patients

We conducted a nonrandomized clinical study at Nancy University Hospital from November 2017 to August 2018. Forty-one patients with primary RD requiring vitrectomy and 33 control patients undergoing vitrectomy for vitreomacular traction (VMT) or macular hole (MH) were included in this study.

Exclusion criteria were any history of vitreoretinal surgery on the eye studied, diabetic retinopathy, or uveitis.

All patients underwent a detailed ophthalmologic examination before surgery, including best-corrected visual acuity measured with projected-light Snellen charts, axial length measurement using IOLMaster (Carl Zeiss Meditec, Dublin, CA), biomicroscopy with anterior segment evaluation, fundus, and careful peripheral retina examination. For the RD group, an Amsler-Dubois scheme was systematically established for each patient, specifying the extent of the RD, number, type and location of retinal breaks, existence of vitreous hemorrhage, and preoperative proliferative vitreoretinopathy (PVR) grading according to Machemer et al. [29].

All patients underwent a three-port 23- or 25-gage pars plana vitrectomy. At the beginning of vitrectomy, air perfusion was set to open and undiluted vitreous fluid samples (1 mL) were collected from each eye with 3 mL syringe. Samples were sent to the Biological Resource Center (Centre de Ressources biologiques, Nancy, France) within 30 min, cooled on ice and transferred into microfuge tubes. Each sample was centrifuged at 10000 × *g* for 5 min and the supernatant was then collected and frozen at − 80 °C before analysis.

### Multiplex bead immunoassay

Inflammatory cytokine concentrations were measured using Bio-Plex Pro™ Human Cytokine 27-plex Assay (Bio-Rad Laboratories, Marnes-la-Coquette, France). Briefly, vitreous samples were thawed and diluted twofold through the use of the dilution solution provided by the Bio-Plex beads array kit. Cytokine levels were measured in duplicate with 50 μL of diluted supernatant in accordance with the manufacturer’s instructions. The following 27 cytokines and chemokines were targeted: IL-1β, IL-1 receptor antagonist (IL-1ra), IL-2, IL-4, IL-5, IL-6, IL-7, IL-8 [chemokine C-X-C motif ligand (CXCL)8], IL-9, IL-10, IL-12, IL-13, IL-15, IL-17, eotaxin [chemokine C-C motif ligand (CCL)11], basic fibroblast growth factor (b-FGF), granulocyte colony-stimulating factor (G-CSF), granulocyte/macrophage colony-stimulating factor (GMCSF), interferon (IFN)-γ, interferon-inducible 10-kDa protein [IP-10 (CXCL10)], MCP-1 (CCL2), macrophage inflammatory protein-1 [MIP-1α (CCL3)], MIP-1β (CCL4), platelet-derived growth factor (PDGF), regulated upon activation, normal T cell expressed and secreted [RANTES (CCL5)], tumor necrosis factor (TNF)-α, and VEGF (vascular endothelial growth factor).

### Mouse model of RD

Wild-type (C57BL/6 J) mice were purchased from Janvier Labs at the age of 8 weeks. Mice were housed in the animal facility under specific pathogen-free condition, in a 12-h/12-h light/dark (100–500 lux) cycle with water and normal diet food available ad libitum. All experimental protocols and procedures were approved by the local animal care ethics committee (N°APAFIS#5201-20160427103344).

RD was induced with a previously described method [12–14]. Briefly, mice were anesthetized with an intraperitoneal injection of xylazine hydrochloride (10 mg/kg) and ketamine hydrochloride (100 mg/kg) and pupils were dilated with topical phenylephrine (5%) and tropicamide (0.5%). A 30-gage needle was first used to create two sclerotomies 1.5 mm posterior to the limbus. A glass needle (with an 80-gage manually beveled tip) connected to a Hamilton syringe filled with diluted sodium hyaluronate (Healon GV®, Alcon) was then introduced into the vitreous cavity through one of the sclerotomies. The tip of the needle was finally inserted into the subretinal space through a peripheral retinotomy and 4 μl of diluted sodium hyaluronate containing or not recombinant human TSP1 (100 μg/ml, Biotechne), human insulin (2 IU/ml, Umuline NPH), recombinant IGF-1 (insulin growth factor-1) (200 ng/ml, Biotechne), or metformin (50 mg/ml, Merck Millipore), was gently injected, detaching approximately two thirds of the retina from the underlying RPE.

For treatment with rosiglitazone, mice received intraperitoneal injections of 10 mg/kg rosiglitazone or vehicle (5% DMSO) 3 days before and 4 to 7 days after RD induction.

Eyes with subretinal hemorrhage were excluded from analysis. Mice were sacrificed from 1 to 10 days following RD, according to the experiment

### Retinal explants culture

C57BL/6 J mouse retina were prepared and placed on polycarbonate filters floating on Dulbecco’s modified Eagle’s medium (DMEM, Thermo Fisher Scientific) with the photoreceptors facing down. For the experiment, retinal explants were cultured in high glucose (25 mM) DMEM alone or supplemented with human insulin (Umuline NPH), human insulin, and insulin receptor inhibitor (Hydroxy-2-naphthalenylmethylphosphonic acid (HNMPA), Abcam) or vehicle (DMSO (dimethyl sulfoxide)) at 37 °C. As insulin has been demonstrated to be quite unstable in media containing cysteine [30], and porcine/human insulin (identical except one amino acid at the C-terminus of the beta chain) is significantly less effective in rodents [31], we used supraphysiological doses of insulin (5 mIU/ml) in our model. For this reason, HNMPA was used at a concentration 10-fold higher than the IC50 (1 mM). Each culture medium was renewed every 36 h and after 5 days, the retinal explants were carefully removed. Immunohistochemistry and cones quantification were then performed as described for retina below.

For monocyte (Mo)/retinal co-cultures, peripheral blood mononuclear cells (PBMCs) were isolated from heparinized venous blood from healthy volunteer individuals. In accordance with the Declaration of Helsinki, volunteers provided written and informed consent for the human monocyte expression studies, which were approved by the Centre National d’Ophthalmologie des Quinze-Vingt Hospital (Paris, France) ethics committees (no. 913572). PBMCs were isolated from blood by 1-step centrifugation on a Ficoll Paque layer (GE Healthcare) and sorted with EasySep Human Monocyte Enrichment Cocktail (StemCells Technology). Human Mos were seeded on polycarbonate filters floating on DMEM for 2 h. C57BL/6 J mouse retina were prepared and placed with the photoreceptors facing 100 000 adherent Mos for 18 h at 37 °C.

### Cone-enriched cultures from chicken embryos

Chick retina cells were isolated and cultured as previously described with minor modifications [32]. Briefly, retinas of 6-day-old chick embryos corresponding to stage 29 were chopped into small fragments and digested using trypsin to prepare single cell solutions. Cells were mechanically dispersed, centrifuged, and suspended in a mixture of 1/1 (vol./vol.) M199 and DMEM, with a final concentration of the following supplements: 100 mg/ml linoleic acid/BSA, 0.86 mM insulin, 0.07 mM transferrin, 2.0 mM progesterone, 0.28 mM prostaglandin, 0.29 mM Na_2_SeO_3_, 182 mM putrescine, 3 mM taurine, 4.7 mM cytidine 50-diphosphocholin, 2.7 mM cytidine 50-diphosphoethanol-amine, 0.55 mM hydrocortison, 0.03 mM triiodo-L-thyronine, 1 mM sodium pyruvate, and 20 mM gentamycin. After incubation of the cells for 7 days with or without TSP1 (80 nM, Biotechne) the cells were stained using Live/deadTM (Thermofisher) according to the manufacturers instruction and photographed. Live and dead cells were quantified in plates seeded at 1 × 10^5^ cells/cm^2^ and at 2 × 10^5^ cells/cm^2^. For each plate cone survival was calculated for 4 TSP1-treated wells versus the average cell number in 14 wells of the negative controls. The protective effect of the cones by molecules is scored as the ratio between the average cell number.

### Isolation of retinal immune cells and flow cytometry analyses

Retinas were dissected out and homogenized in 500 μL of PBS (phosphate-buffered saline) with liberase TL at 0.8 wunsch/mL (Sigma-Aldrich) for 30 min at 37 °C and 5% CO_2_. The retinal homogenate was washed with PBS and the pellet containing the immune cells was re-suspended in 100 μL PBS containing 1 μL of Viobility 405/520 Fixable Dye (Miltenyi). Cells were washed and labeled with 50 μL of primary antibodies mix: anti-CD45-VioBlue (REA737), anti-MHCII-FITC (REA813), anti-CD11b-PE (REA592), anti-Ly-6C-PE-Vio770 (REA796), anti-CD3-APC (REA641), and anti-Ly-6g-APC-Vio770 (REA526) (Miltenyi). After labeling, cells were fixed in 4% paraformaldehyde. For compensation settings, single-stained cellular controls with corresponding antibodies were used. Fluorescence intensities were measured using a MACSQuant analyzer (Miltenyi) and data were analyzed using the FlowJo Software.

### Immunohistochemistry of retinal flatmounts

Eyes were enucleated, fixed in 4% paraformaldehyde for 1 h at room temperature and sectioned at the limbus; the cornea and lens were discarded. The retinas were peeled from the RPE/choroid/sclera and incubated overnight at 4 °C in PBS-1% triton with the following primary antibodies: peanut agglutinin (PNA) Alexa fluor® 594 (Thermo Fisher Scientific; 1/100), rabbit polyclonal anti-human cone arrestin (CAR) antibody (LUMIF-hCAR; 1:10000), and goat polyclonal anti-IBA1 (ionized calcium-binding adapter molecule-1) (Abcam; 1:100). After a few washes, the retinas were incubated for 2 h at room temperature with appropriate Alexa Fluor® conjugated secondary antibodies (Thermo Fisher Scientific; 1:500) in PBS-1% triton and nuclei were counterstained with Hoechst (1:1000, Sigma-Aldrich). The retinas were flatmounted and viewed with a fluorescence microscope (DM5500, Leica). Images centered on the area with the lowest number of PNA+ cone arrestin+ cells were captured with a confocal laser-scanning microscope (FV1000, Olympus) using a × 40 lens. Each cell population was manually counted in a masked fashion. IBA-1+ cells were quantified on flatmounts on the outer segment side of the detached retina while PNA+ cone arrestin+ cells were counted on confocal microscopy Z-stacks using ImageJ software.

### Reverse transcription and real-time quantitative polymerase chain reaction

Total RNA was extracted from mouse retina with the Nucleospin RNAII extraction kit according to the manufacturer’s protocol (Macherey Nagel). Single-stranded cDNA was synthetized from total mRNA (pretreated with DNase) using oligo-dT as primer and superscript II reverse transcriptase (Thermo Fisher Scientific). Subsequent RT-PCR was performed using cDNA, PowerSYBR Green PCR Master Mix (Applied Biosystems) and primers (IDT technology) available upon request. qPCR was performed using StepOne Plus real-time PCR systems (Applied Biosystems) with the following profile: 45 cycles of 15 s at 95 °C, 45 s at 60 °C. Results were normalized using house-keeping gene RPS26.

### Statistical analysis

Graph Pad Prism 7 (GraphPad Software) was used for data analysis and graphic representation. All values are reported as mean ± SEM. Statistical analyses were performed by one-way Anova (analysis of variance), Student *t* test, or Mann-Whitney *U* test for comparison among means depending on the experimental design. The *p* values are indicated in the figure legends.

## Results

### RD causes a marked inflammatory response with increased cytokine and chemokine expression in both human and experimental models

**Fig. 1:**
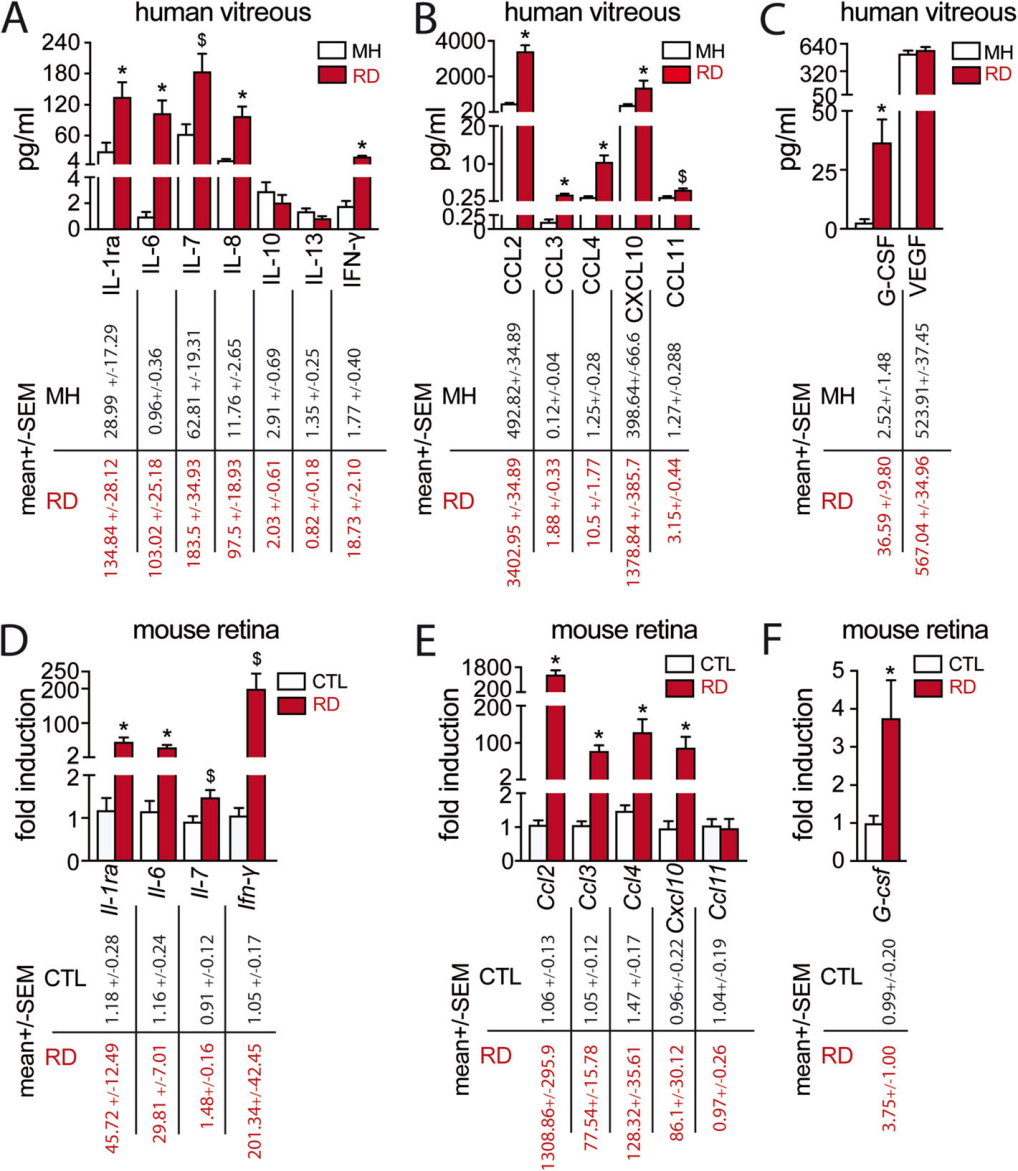
RD causes a marked inflammatory response with increased cytokine and chemokine expression in both human and experimental models. Vitreous concentrations of **a** cytokines, **b** chemokines, and **c** growth factors from MH and RD groups of patients (34 MH and 41 RD samples; **a** ***t*** test or Mann-Whitney ***U*** test, *p < 0.0001 and ^**$**^***p*** = 0.009 versus control; **b** ***t*** test or Mann-Whitney ***U*** test, *p < 0.0001 and ^**$**^***p*** = 0.002 versus control; **c** ***t*** test *p = 0.002 versus control). Relative expression of **d** cytokine, **e** chemokine, and **f** growth factor mRNAs normalized with S26 expression quantified by RT-qPCR in mouse retina without RD and 4 days after RD (***n*** = 6–8/group; Mann-Whitney ***U*** test *p < 0.01 and ^**$**^***p*** = 0.03 versus control). RD, retinal detachment; MH, macular hole. All values are reported as mean ± SEM

To characterize the inflammatory response to RD, we first analyzed the expression profile of cytokines in vitreous samples from 41 patients with RD and from 34 control patients with macular hole. The mean extent of detachment in the RD group was 2.1 ± 0.8 quadrants with a macular involvement in 34.1% of cases and some degree of retinal wrinkling and folding (grade B or C proliferative vitreoretinopathy) in 36.6% of cases. The mean duration of symptoms before surgery was 7.7 ± 7.3 days with a median of 4.5 days [1–30] (Table 1). Using a Human Cytokine 27-plex Assay, we showed that the cytokines IL-1ra, IL-6, IL-7, IL-8, and IFN-γ (Fig. 1 a); the chemokines CCL2, CCL3, CCL4, CXCL10, and CCL11 (Fig. 1 b); and the growth factor G-CSF (Fig. 1 c) were significantly increased in the vitreous from RD patients. In contrast, the levels of IL-10, IL-13, and VEGF were not statistically different between the two groups (Fig.1 a, c). The remaining cytokines, such as IL-4, and IL-17 of the assay were not detectable.

**Table 1:** Baseline characteristics of patients who underwent vitrectomy for retinal detachment (RD group) and macular hole or vitreomacular traction (control group)

	RD group	Control group
**Number of eyes/patients**, *n*	41	33
**Age**, years (mean ± SD)	62.4 ± 12.4	72.2 ± 9.1
**Male**, *n* (%)	27(65.9)	7(21.2)
**Duration of RD**, days (mean ± SD)	7.7 ± 7.3	-
**Extent of RD**, quadrants (mean ± SD)	2.1 ± 0.8	-
**Macular status**		
Macula on*, n* (%)	14(34.1)	-
Macula off*, n* (%)	27(65.9)	-
**Full-thickness MH**, *n* (%)	0	24(72.7)
**Vitreomacular traction without MH**, *n* (%)	0	9(27.3)

*SD* standard deviation, *RD* retinal detachment, *MH* macular hole

We next evaluated the expression of the cytokines by RT-qPCR of mouse retinal control tissues and retinas harvested after 4 days of experimental RD. This time point was chosen for analysis as it was similar to the median duration of symptoms in our clinical study and because photoreceptor cell death peaks at around 3 days after RD in both experimental models and human samples [4, 33–35]. The mRNA levels of nine out of eleven mediators found to be elevated in human vitreous from RD patients (except for *Ccl11* and *Il-8* that does not exist in mice*)* were significantly upregulated in detached mouse retinas compared to controls (Fig. 1 d–f).

In summary, our findings confirm that RD induces a marked inflammatory response in human patients, which is closely mimicked by experimental murine retinal detachment. The cytokine profile is suggestive of an infiltration of mainly myeloid cells, but the increased levels of IFN-γ might be suggestive of T helper type 1 (Th1) cells recruitment.

### RD-associated leukocyte infiltration is associated with cone loss

**Fig. 2:**
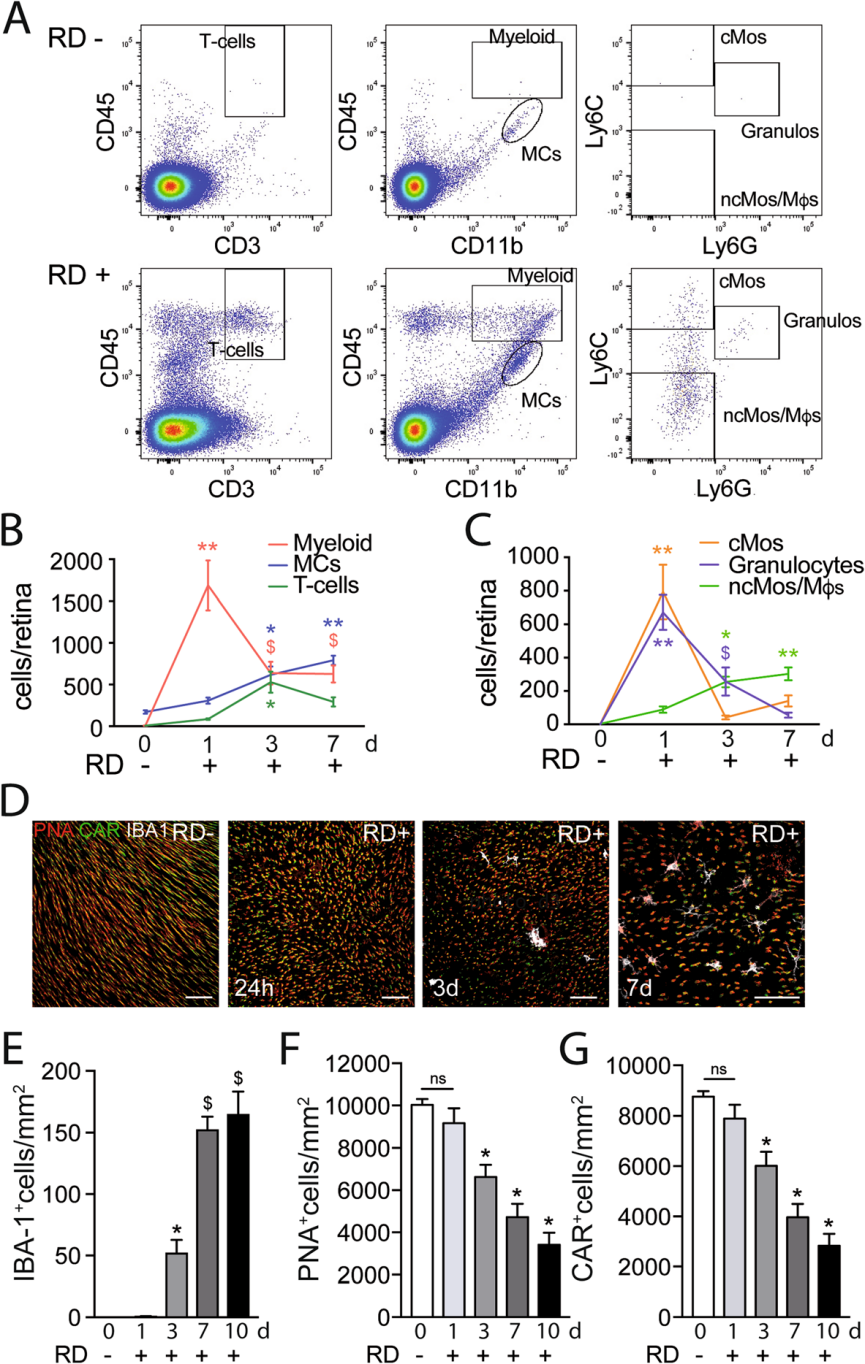
RD-associated leukocyte infiltration is associated with cone loss. **a** Representative cytometry plots of mouse retina without RD (upper panels) or 7 days after RD (lower panels). CD45^+^CD3^+^ cells define T cells and CD45^+^CD11b^+^ cells define microglia and myeloid cells with CD45^low^CD11b^+^ representing microglia and CD45^high^CD11b^+^ representing infiltrating myeloid cells. CD45^high^CD11b^+^ were further defined with Ly6C and Ly6G with Ly6C^high^Ly6G^-^ cells representing monocytes, Ly6C^low^Ly6G^-^ representing macrophages and Ly6G^+^ representing granulocytes. Flow cytometry quantification of **b** T cells, MCs, and infiltrating myeloid cells and **c** Mos, Mφs, and neutrophils in mouse retina without RD and 24 h, 3 days, and 7 days after RD (***n*** = 8–16/group; **b** one-way Anova *p < 0.006, **p < 0.0001, and ^**$**^***p*** < 0.05 versus the control group; **c** one-way Anova *p = 0.0016, **p < 0.0001, and ^**$**^***p*** = 0.0384 versus the control group). **d** Representative images of the subretinal aspect of retinal flatmounts of peanut agglutinine- (PNA, red), cone arrestin- (CAR, green), and ionized calcium-binding adapter molecule 1- (IBA-1, white) stained retina of C57BL6/J mice without RD, 24 h, 3 days, and 7 days after RD. Quantification of **e** subretinal IBA-1+ cells, **f** PNA+ cones, and **g** CAR+ cones in retinal flatmounts of C57BL6/J mice without RD and 1 day, 3 days, 7 days, and 10 days after RD (***n*** = 6–12/group; **e** one-way Anova *p = 0.0153 and ^**$**^***p*** < 0.0001 versus the control group; **f** one-way Anova *p < 0.0001 versus the control group; **g** one-way Anova *p < 0.0001 versus the control group). RD, retinal detachment; Myeloid, myeloid cells; cMos, classical monocytes; MCs, microglial cells; ncMos/Mφs, non-classical monocytes/macrophages; Neutros, neutrophils; PNA, peanut agglutinine; CAR, cone arrestin; IBA-1, ionized calcium-binding adapter molecule 1. Scale bar (**d)** = 50 μm. All values are reported as mean ± SEM

In the healthy retina, microglial cells (MCs) populate the inner retina, but the photoreceptor cell layer and subretinal space are devoid of any immune cells [36]. In RD, MPs have been shown to accumulate in the detached area and are highly associated with TUNEL-positive nuclei in the inner aspects of the outer nuclear layer (ONL), where rod nuclei are located [11, 13, 14]. Using flow cytometry and a gating strategy (Fig. 2 a) adapted from O’Koren et al. [37], we here analyzed the leukocyte population in healthy and detached (day 1, day 3, day 7) mouse retinas. In healthy retinas, we only detected CD11b^+^CD45^med^MHCII^-^MCs (that were also proofed to be CD11c^-^ in separate cytometric analysis). After RD, this number steadily increased to quadruple at the end of the observation period at day 7 (Fig. 2 b). The CD11b^+^CD45^high^ myeloid cell population sharply increased after RD, peaking at 24 h and remained strongly elevated throughout (Fig. 2 b). Interestingly, we also found a sizeable population of CD45^+^CD3^+^- T cells. These cells infiltrated the retina at day 3 and remained elevated, although to a lesser extent, at day 7 (Fig. 2 b). A more detailed analysis of the myeloid population reveals a rapid but short-lived recruitment of CD11b^+^CD45^high^Ly6G^+^ granulocytes and CD11b^+^CD45^high^Ly6G^neg^Ly6C^high^ classical monocytes (cMos) at day 1 (Fig. 2 c). The number of CD11b^+^CD45^high^Ly6G^neg^Ly6C^low^ cells that represent Mo-derived macrophages and non-classical monocytes, increased at day 3 and stayed elevated (Fig. 2 c). Together, these results demonstrate that RD induces the infiltration and accumulation of T cells, Mos, and Mo-derived-macrophages (Mφs) and MCs.

Next, we quantified the presence of MPs (MCs and Mφs) and the cone population on ionized calcium-binding adapter molecule-1 (IBA-1), also called allograft inflammatory factor 1 that is strongly expressed by MPs {immgen.org} and commonly used as an MP marker [38], peanut agglutinin (PNA cone outer segment marker), cone arrestin (CAR, cone marker) triple-stained retinal flatmounts (Fig. 2 d). Confocal microscopy confirmed that subretinal MPs are not observed in normal mice and highlight the elongated shape of the cone outer segments (Fig. 2 d). Interestingly, despite the peak of infiltrating myeloid cells measured in the whole retina at day 1, IBA-1^+^MPs only accumulated in the subretinal space by day 3 and continued to rise to reach a plateau at day 7 (Fig. 2 e), similar to previous reports [7]. Although cone outer segments seemed shortened at day 1 (Fig. 2 d), their number only decreased significantly in the following days and was reduced by approximately 50% at day 7 (Fig. 2 d, f, g) mirroring the subretinal MPs accumulation.

Taken together, our results demonstrate that RD leads to a rapid infiltration of myeloid cells, followed by T cells and a protracted increase of the numbers of MCs and Mφs that started accumulating in the subretinal space by day 3. We also showed that this accumulation was strongly associated with cone death.

### TSP1 inhibits RD-induced subretinal MP infiltration and associated cone loss

**Fig. 3:**
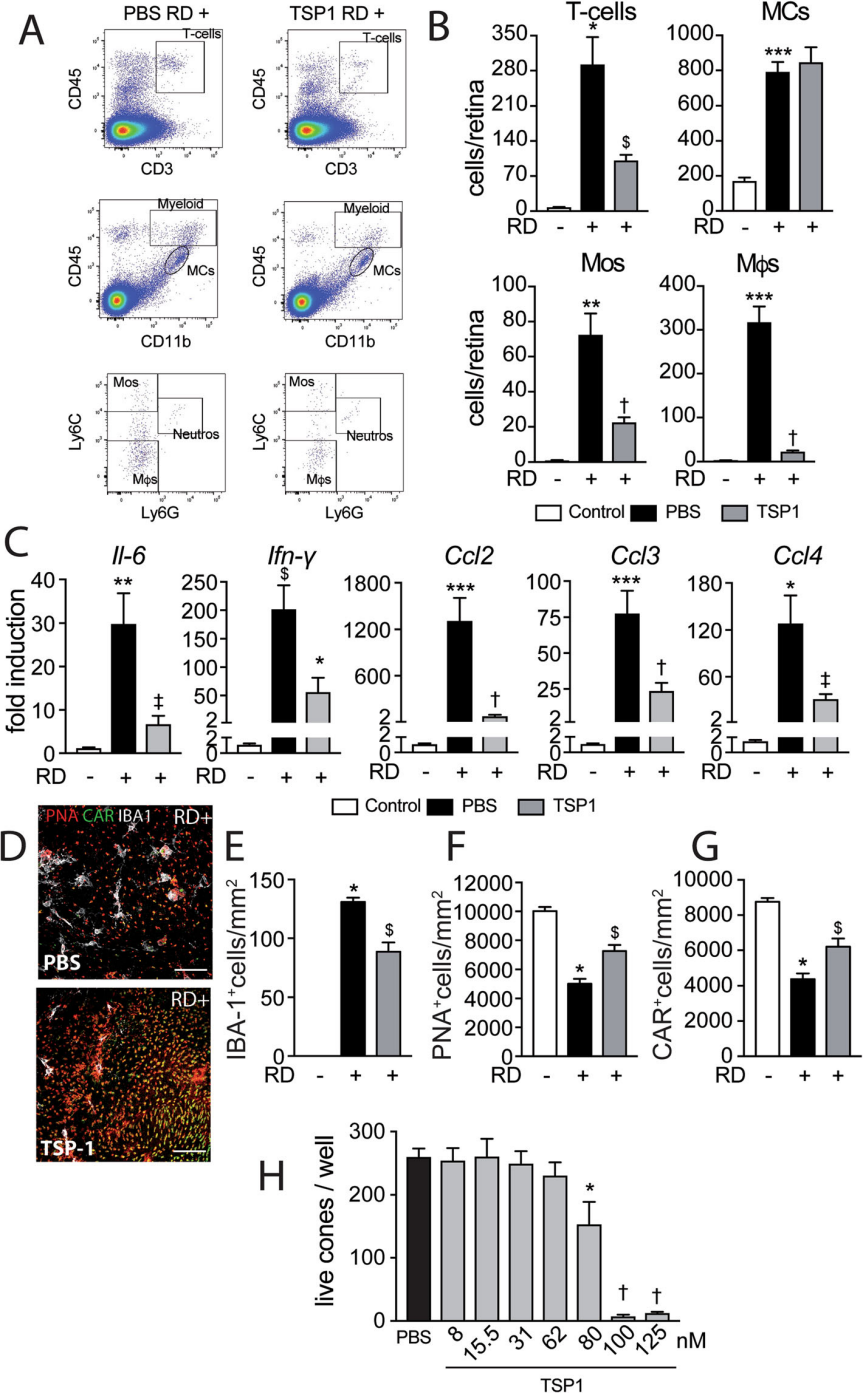
TSP1 inhibits RD-induced leukocyte infiltration and prevents cone loss. **a** Representative cytometry plots of mouse retina 7 days after RD without (upper panels) or with subretinal injection of TSP1 (lower panels). CD45^+^CD3^+^ cells define T cells and CD45^+^CD11b^+^ cells define microglia and myeloid cells with CD45^low^CD11b^+^ representing microglia and CD45^high^CD11b^+^ representing infiltrating myeloid cells. CD45^high^CD11b^+^ were further defined with Ly6C and Ly6G with Ly6C^high^Ly6C^-^ cells representing monocytes, Ly6C^low^Ly6C^-^ representing macrophages and Ly6G^+^ representing neutrophils. **b** Flow cytometry quantification of T cells, MCs, Mos, and Mφs in mouse retina without RD and 7 days after RD without or with subretinal injection of TSP1 (***n*** = 8–24/group; one-way Anova/Bonferroni test *p = 0.0018, **p = 0.0003, and ***p < 0.0001 versus the control group, ^**$**^***p*** = 0.0032 and †***p*** < 0.0005 versus the PBS group). **c** Quantitative RT-PCR of Il-6, Ifn-γ, Ccl2, Ccl3, and Ccl4 mRNAs normalized to S26 mRNA in mouse retina without RD and 4 days after RD with or without subretinal injection of TSP1 (***n*** = 6–8/group; one-way Anova/Bonferroni test *p < 0.003, **p < 0.001, and ***p < 0.0001 versus the control group, ^**$**^***p*** < 0.01, ^**‡**^***p*** < 0.007, and †***p*** < 0.0005 versus the PBS group). **d** Representative images of peanut agglutinine- (PNA, red), cone arrestin- (CAR, green), and ionized calcium-binding adapter molecule 1- (IBA-1, white) stained retinal flatmounts of C57BL6/J mice 7 days after RD without or with subretinal injection of TSP1. Quantification of **e** subretinal IBA-1+ cells, **f** PNA+ cones, and **g** CAR+ cones in retinal flatmounts of C57BL6/J mice without RD and 7 days after RD without or with subretinal injection of TSP1 (***n*** = 6–12/group; **e** one-way Anova/Bonferroni test *p < 0.0001 versus the control group, ^**$**^***p*** = 0.0002 versus the PBS group; **f** one-way Anova/Bonferroni test *p < 0.0001 versus the control group, ^**$**^***p*** = 0.0470 versus the PBS group; **g** one-way Anova/Bonferroni test *p < 0.0001 versus the control group, ^**$**^***p*** = 0.0448 versus the PBS group). **h** Live cone counts per well of cone-enriched cultures from chicken embryos treated with PBS or the indicated TSP1 concentration for 7 days (***n*** = 15 for PBS and ***n*** = 4 for each TSP1 concentration one-way Anova/Bonferroni test *p = 0.0219, †***p*** < 0.0001 versus PBS). RD, retinal detachment; PBS, phosphate-buffered saline; TSP1, thrombospondin-1; Myeloid, myeloid cells; Mos, monocytes; MCs, microglial cells; Mφs, macrophages; Neutros, neutrophils; PNA, peanut agglutinine; CAR, cone arrestin; IBA-1, ionized calcium-binding adapter molecule 1. Scale bar (**d)** = 50 μm. All values are reported as mean ± SEM

TSP1 is an extracellular matrix molecule that is produced by a wide variety of cell types, notably the RPE, inflammatory, and resident macrophages [36]. We and others have shown that it physiologically prevents age-related subretinal MP accumulation and inhibits excessive subretinal MP infiltration and choroidal neovascularization in the context of age-related macular degeneration [39–41] and controls T cell responses [42]. To further explore the role of infiltrating MPs in RD-associated cone loss, we induced RD with 4 μl of diluted sodium hyaluronate that contained or not recombinant TSP1 (100 μg/ml). Considering that 4 ml correspond to roughly 1/10 of the 40-μl sized adult mouse eye, we estimate the efficient intraocular TSP1 concentration to be 10 μg/ml (80 nM). Using flow cytometry and the same gating strategy as in Fig. 2 (Fig. 3 a), we found that recombinant TSP1 significantly reduced the number of T cells, Mos, and Mφs but had no effect on the MC population (Fig. 3 b). Accordingly, RT-qPCR analysis showed that recombinant TSP1 significantly reduced the mRNA levels of *Il-6*, *Ifn-*γ*, Ccl2*, *Ccl3,* and *Ccl4* (Fig. 3 c). Quantification of IBA-1^+^MPs and PNA^+^CAR^+^cones on triple-stained retinal flatmounts at day 7 (Fig. 3 d) showed that recombinant TSP1 also significantly decreased subretinal MP accumulation (Fig. 3 e) and increased cone survival in detached retinas compared with PBS controls (Fig. 3 f, g).

To test whether TSP1 directly affects cone survival, we treated cone-enriched cultures from chicken embryos for 7 days with either PBS or recombinant TSP1. This model was developed to identify cone viability factors such as RdCVF, which significantly inhibits cone death that occurs over the 7 days of culture, doubling the number of live cones/well at d7 [16, 32]. Chicken and human and mouse and human TSP1 are 85% and 84% identical, respectively (https://blast.ncbi.nlm.nih.gov/Blast.cgi). Furthermore, chicken, mouse, and human TSP1 carry the same CSVTCG CD36 binding sequences and the two VVM sites that bind the CD47 receptors of TSP1. Functionally, human purified TSP1 inhibits proliferation of human dermal microvascular endothelial cells and chicken microvascular brain endothelial cells equally [43], showing that human TSP1 activates chicken (and mouse) TSP1 receptors equally.

Quantification of calcein^+^ viable cones in live/dead^TM^-stained culture wells after 7 days of culture revealed that TSP1 did not increase cone survival in the culture. On the contrary, TSP1 at 80 nM (10 μg/ml), the concentration that corresponds to the in vivo experiments (see above) and that induces Mo death [44] and inhibits chicken and human endothelial cell proliferation in vitro [43], and concentrations above significantly reduced the number of live cone cells at day 7 of the culture (Fig. 3 h).

Together, our findings that TSP1 reduces the number of infiltrating immune cells and increases cone survival in RD but does not directly increase cone survival in cone-enriched cultures strongly suggest that it is TSP1s ability to inhibit immune cell infiltration in RD that protects against cone loss. Infiltrating immune cells would thereby contribute to cone loss similar to previously described inflammation-induced rod loss in RD [11, 13, 14].

### RD-associated inflammation inhibits RdCVF expression

Cones are highly dependent on glucose uptake for survival [16, 17], which is regulated by RdCVF [16]. Interestingly, a transcriptomic analysis of human long-term detached retina showed an association of increased inflammatory markers and decreased RdCVF expression [45].

**Fig. 4:**
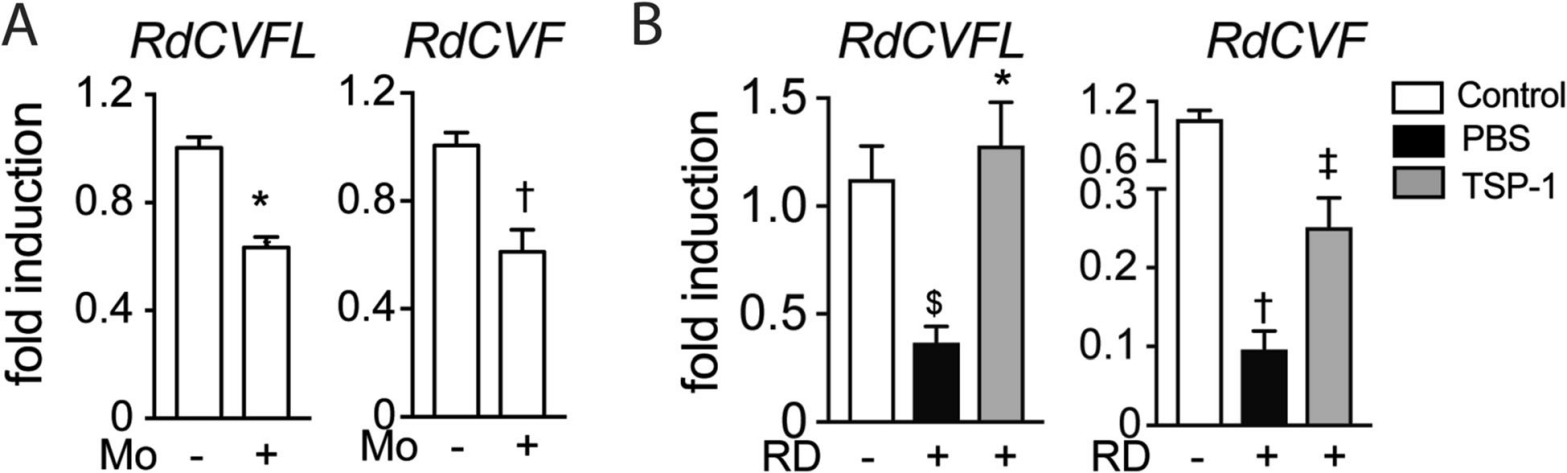
RD-associated inflammation inhibits RdCVF expression. Quantitative RT-PCR of *RdCVFL* and *RdCVF* mRNAs normalized to S26 mRNA in **a** retinal explants cultured for 18 h with or without human monocytes (***n*** = 6; Mann-Whitney *p and †***p*** = 0.0022) and **b** mouse retina without RD and 4 days after RD with or without subretinal injection of TSP1 (***n*** = 6–10/group; one-way Anova/Bonferroni test ^$^***p*** = 0.006, †***p*** < 0.0001 versus the control group, *p = 0.001 and ^**‡**^***p*** = 0.046 versus the PBS group; Mann-Whitney test versus the PBS group *p = 0.0006 and ^**‡**^***p*** = 0.0021)

To test whether immune cells affect retinal RdCVF expression, we first analyzed its expression in 18 h C57BL/6 mouse retinal explants that we co-cultured with 100,000 human blood-derived CD14^+^Mos adhering to polycarbonate filters floating on DMEM (with the photoreceptors facing the adherent Mos). This short-term retinal explant model induces the apoptosis of 2–3% of rod photoreceptors (1500 apoptotic rod nuclei/mm2 containing approximately 60,000 rods) and a severe loss of cone outer segments [46]. RT-qPCR analysis revealed a significant reduction in RdCVFL- (the thioredoxin containing long form) and RdCVF-mRNA in explants that were co-cultured with Mos compared to retinal explant mono-cultures (Fig. 4 a). In vivo, 4 days of RD severely reduced the expression levels of RdCVFL and RdCVF by 67% and 90%, respectively (Fig. 4 b). TSP1, which inhibits the accumulation of MPs and T cells (Fig. 3), increased the expression of RdCVFL in detached retina by 350% to normal levels and increased RdCVF expression by 260 to 25% of the expression of undetached retina. The decrease in the expression of RdCVFL (expressed by rods and cones) and RdCVF (exclusively produced by rods [32]) could not be attributed to rod loss (as observed in retinitis models) as the majority of rods are still present in 4-day RD and 18 h retinal explants cultured with Mos.

Taken together, the fact that Mos decrease RdCVF/RdCVFL expression in explants and that TSP1, which inhibits MP and T cell accumulation, significantly improves RdCVF/RdCVFL expression in RD, strongly suggests that RD-associated inflammation contributes to the reduction of RdCVF expression in RD. In RD, cone glucose availability could therefore become critical as highly metabolic active immune cells compete for fuel in the photoreceptor cell layer [27] and RD-induced inflammation reduce RdCVFL and RdCVF and further hamper glucose uptake specifically in cones.

### Insulin delays RD-induced cone loss in vivo

In retinitis pigmentosa, cones die secondarily to the loss of rods and the collapse of RdCVF levels that is physiologically secreted by rods and regulates cone glucose uptake [32]. Administration of insulin improves glucose uptake in cones and delayed their death, despite the absence of RdCVF [19, 20]. As recombinant RdCVF is not available, we investigated whether insulin could improve cone survival in the context of RD.

**Fig. 5:**
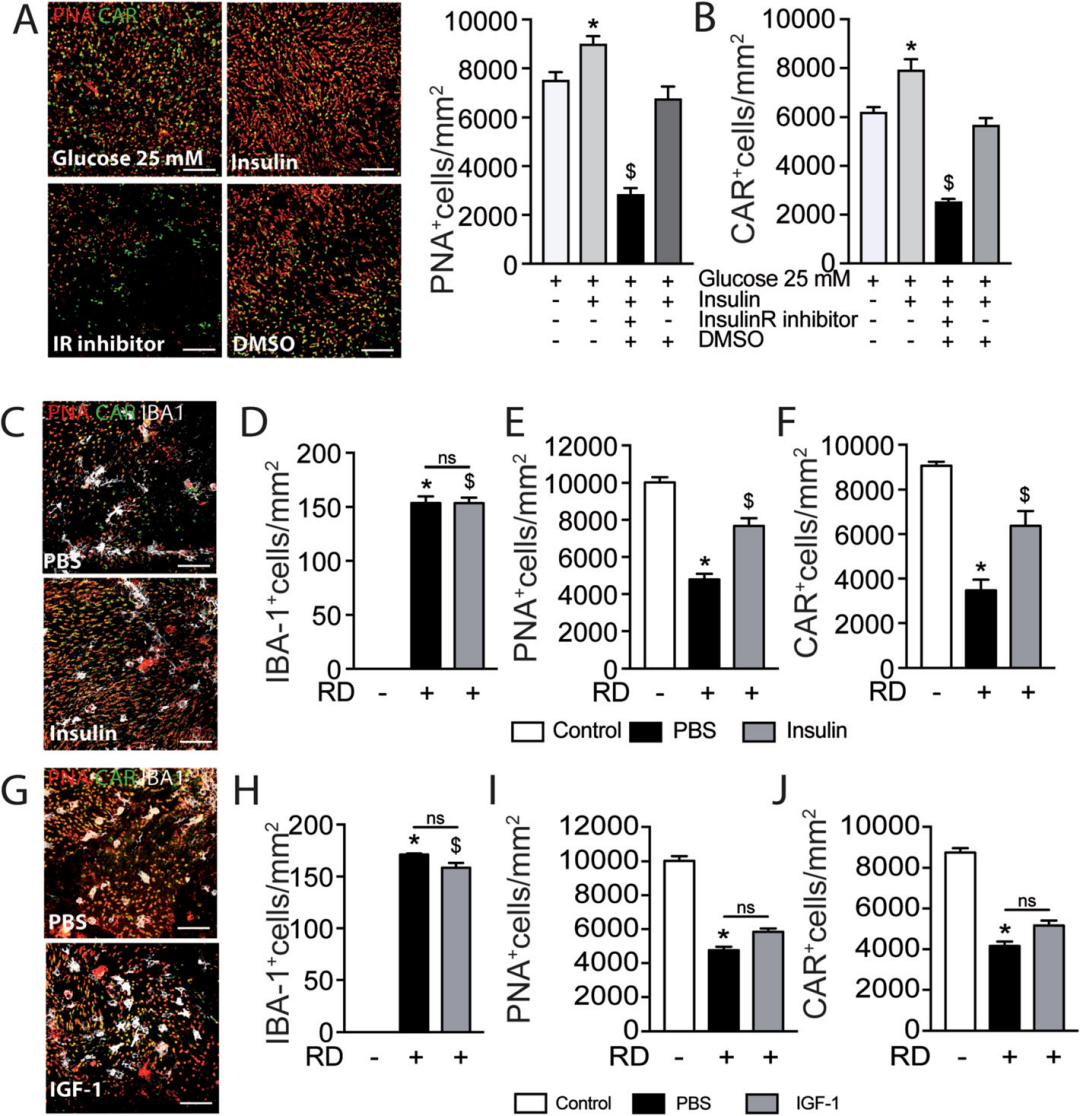
Insulin is essential for cone survival **in vitro** and delays RD-induced cone loss **in vivo***.* Representative images of peanut agglutinine- (PNA, red) and cone arrestin- (CAR, green) stained retinal explants and quantification of **a** PNA+ cones and **b** CAR+ cones after 5 days of culture in high glucose DMEM (25 mM) with or without human insulin and/or insulin receptor inhibitor (HNMPA) or its vehicle (***n*** = 5/group; **b** one-way Anova *p = 0.0358 and ^**$**^***p*** < 0.0001 versus the high glucose culture; **c** one-way Anova *p = 0.0012 and ^**$**^***p*** < 0.0001 versus the high glucose culture). **c** Representative images of peanut agglutinine- (PNA, red), cone arrestin- (CAR, green) and ionized calcium-binding adapter molecule 1- (IBA-1, white) stained retinal flatmounts of C57BL6/J mice 7 days after RD without or with subretinal injection of insulin. Quantification of **d** subretinal IBA-1+ cells, **e** PNA+ cones, and **f** CAR+ cones in retinal flatmounts of C57BL6/J mice without RD and 7 days after RD with or without subretinal injection of insulin (***n*** = 6–12/group; **e** one-way Anova *p < 0.0001 and ^**$**^***p*** < 0.0001 versus the control group; **f** one-way Anova/Bonferroni test *p < 0.0001 versus the control group, ^**$**^***p*** = 0.0020 versus the PBS group; **g** one-way Anova/Bonferroni test *p < 0.0001 versus the control group, ^**$**^***p*** = 0.0002 versus the PBS group). **g** Representative images of peanut agglutinine- (PNA, red), cone arrestin- (CAR, green), and ionized calcium-binding adapter molecule 1- (IBA-1, white) stained retinal flatmounts of C57BL6/J mice 7 days after RD without or with subretinal injection of IGF-1. Quantification of **h** subretinal IBA-1+ cells, **i** PNA+ cones, and **j** CAR+ cones in retinal flatmounts of C57BL6/J mice without RD and 7 days after RD with or without subretinal injection of IGF-1 (***n*** = 6–12/group; **g** one-way Anova *p < 0.0001, ^**$**^***p*** < 0.0001 versus the control group; **h** one-way Anova *p < 0.0001 versus the control group; **i** one-way Anova *p < 0.0001 versus the control group). PNA, peanut agglutinine; CAR, cone arrestin; IR, insulin receptor; DMSO, dimethyl sulfoxide; IBA-1, ionized calcium-binding adapter molecule 1; PBS, phosphate-buffered saline; RD, retinal detachment; IGF-1, insulin-like growth factor; DMEM, Dulbecco’s modified Eagle’s medium; HNMPA, hydroxy-2-naphthalenylmethylphosphonic acid. Scale bar (**a**, **c**, **g)** = 50 μm. All values are reported as mean ± SEM

We first examined the effect of insulin on 5 day retinal explants with or without human insulin and/or the specific insulin receptor inhibitor HNMPA (Fig. 5 a). Quantification of PNA^+^CAR^+^ cones showed that cone survival was significantly increased in the presence of insulin in the medium culture compared to the control condition (Fig. 5 a, b). The addition of an insulin receptor inhibitor HNMPA that blocks insulin receptor autophosphorylation, but not insulin growth factor 1 (IGF-1) receptor activation [47], resulted in a severe loss of PNA^+^CAR^+^ cones, that by far exceeded the toxicity of its vehicle (DMSO) (Fig. 5 a, b). Our results indicate that insulin signaling promotes cone survival in retinal explants confirming previous results in a mouse retinal degeneration model [19, 20].

Next, we induced RD in vivo with subretinal injection of diluted sodium hyaluronate containing (or not) human insulin (2 IU/ml). Quantification on immuno-stained retinal flatmounts at day 7 (Fig. 5 c) revealed that insulin treatment did not alter the numbers of subretinal IBA1^+^ MPs, but very significantly increased the number of PNA^+^CAR^+^ cones compared to PBS controls (Fig. 5 d–f). Comparatively, addition of IGF-1 to the detachment inducing gel (at a concentration 100-fold higher than IGF-1s ED50, which has been shown to reverse hypoalgesia in diabetic mice [48]) had no effect on the numbers of IBA1^+^ MPs or PNA^+^CAR^+^ cones, quantified on day 7 immuno-stained retinas (Fig. 5 g–j).

Taken together, our results showed that insulin and insulin receptor signaling were essential for cone survival ex vivo of adult retinas and that insulin treatment very significantly inhibited RD-induced cone loss despite the unchanged MPs infiltration in vivo. This effect was not due to insulin-induced IGF-1R signaling, which can activate anti-apoptotic IGF-1 receptor signaling [49, 50], as IGF-1 had no comparable effect.

### The insulin sensitizer rosiglitazone and metformin prevent RD-induced cone loss

Glucose uptake can also be increased by insulin sensitizers, such as rosaglitazone [51, 52]. To explore whether pharmacological improvement of insulin signaling could reduce inflammation-induced cone degeneration in RD, we next examined whether rosiglitazone could prevent cone loss in our mouse model of RD.

**Fig. 6:**
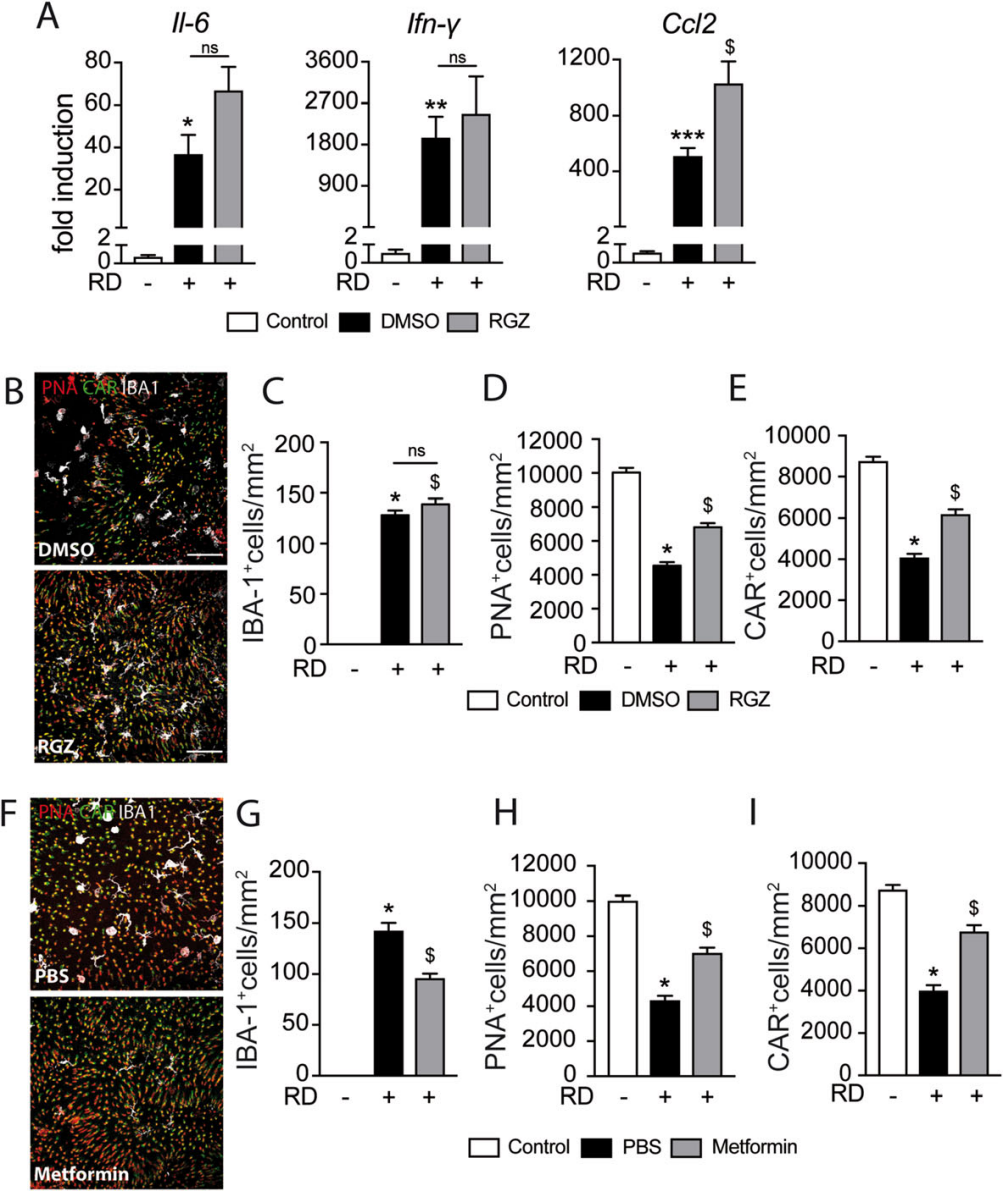
The insulin sensitizer rosiglitazone prevents RD-induced cone loss. **a** Quantitative RT-PCR of Ccl2, Il-6, and Ifn-γ mRNAs normalized to S26 mRNA in mouse retina without RD and 4 days after RD and treatment with rosiglitazone or its vehicle (***n*** = 6–10/group; one-way Anova/Bonferroni test *p = 0.0494, **p = 0.0326, and ***p = 0.0036 versus the control group, ^**$**^***p*** = 0.0028 versus the DMSO group). **b** Representative images of peanut agglutinine- (PNA, red), cone arrestin- (CAR, green), and ionized calcium-binding adapter molecule 1- (IBA-1, white) stained retinal flatmounts of C57BL6/J mice 7 days after RD and treatment with rosiglitazone or its vehicle (DMSO). Quantification of **c** of subretinal IBA-1+ cells, **d** PNA+ cones, and **e** CAR+ cones in retinal flatmounts of C57BL6/J mice without RD and 7 days after RD and treatment with rosiglitazone or its vehicle (***n*** = 6–12/group; **c** one-way Anova *p < 0.0001 and ^**$**^***p*** < 0.0001 versus the control group; **d** one-way Anova/Bonferroni test *p < 0.0001 versus the control group, ^**$**^***p*** = 0.0018 versus the DMSO group; **e** one-way Anova/Bonferroni test *p < 0.0001 versus the control group, ^**$**^***p*** = 0.0031 versus the DMSO group). **f** Representative images of peanut agglutinine- (PNA, red), cone arrestin- (CAR, green), and ionized calcium-binding adapter molecule 1- (IBA-1, white) stained retinal flatmounts of C57BL6/J mice 7 days after RD without or with subretinal injection of metformin. Quantification of **g** subretinal IBA-1+ cells, **h** PNA+ cones, and **i** CAR+ cones in retinal flatmounts of C57BL6/J mice without RD and 7 days after RD without or with subretinal injection of metformin (***n*** = 6–12/group; **g** one-way Anova *p < 0.0001 versus the control group, ^**$**^***p*** < 0.0001 versus the PBS group; **h** one-way Anova *p < 0.0001 versus the control group, ^**$**^***p*** = 0.0056 versus the PBS group; **i** one-way Anova *p < 0.0001 versus the control group, ^**$**^***p*** = 0.0004 versus the PBS group). RD, retinal detachment; DMSO, dimethyl sulfoxyde; RGZ, rosiglitazone; PNA, peanut agglutinine; CAR, cone arrestin; IBA-1, ionized calcium-binding adapter molecule 1. Scale bar (**a**, **b**, **f)** = 50 μm. All values are reported as mean ± SEM

Mice received daily intraperitoneal injections of rosiglitazone or vehicle (DMSO 5%) 3 days before and throughout the 7 days of RD. The treatment also did not alter the increased levels of *Il-6* and *Ifn-γ* mRNA levels in whole retinal mRNA and increased *Ccl2* in 4-day RD samples (Fig. 6 a). Quantification of IBA1-, PNA-, and CAR triple-stained retinal flatmounts (Fig. 6 b) showed that rosiglitazone had no effect on the number of infiltration of subretinal IBA-1^+^ MPs at day 7 (Fig. 6 c). Despite this lack of an anti-inflammatory effect, quantification of PNA^+^CAR^+^ cones revealed that rosiglitazone significantly protected retinas against cone loss at day 7 (Fig. 6 d, e). Interestingly, subretinal injection of metformin, a commonly used insulin sensitizer that also exerts an independent anti-inflammatory effect [53, 54], significantly increased cone survival and decreased subretinal MP accumulation in detached retinas compared with PBS controls (Fig. 6 f–i).

In summary, our results show that the well-established insulin sensitizers rosiglitazone and metformin significantly curb cone loss in RD. The fact that we observed increased cone survival under rosiglitazone and insulin treatment in the absence of an anti-inflammatory effect strongly suggests that increasing insulin signaling was the likely mode of action.

## Discussion

Despite successful surgical repair, visual recovery remains incomplete in many eyes that have suffered from macula-off RD, mainly because of photoreceptor cell death [4, 5, 33], in particular cone loss [15]. Although evidence from both human and experimental studies show that inflammation is strongly associated with RD-induced photoreceptor cell death [7, 11–14], little is known about the mechanisms that specifically lead to cone death.

Our analysis of vitreous samples confirms that RD in humans is associated with increased expression of cytokines, chemokines, and growth factors [6, 7, 9, 55]. Our experimental model also replicates a very similar induction in RD in mice [7], confirming it is well-suited for studying the effect of these mediators on RD-associated cone loss. Using immunohistochemistry and flow cytometry, we corroborate the RD-induced immune cell accumulation [7, 10]. Our flow cytometric analysis showed a rapid infiltration of granulocytes and Mos (day 1), followed by T-lymphocytes (day 3) and a protracted increase of the numbers of MCs and Mφs that were immunohistochemically detected in the subretinal space from day 3 following RD, confirming earlier reports [7].

RD-induced MP accumulation has previously been shown to be highly associated with TUNEL-positive nuclei in the inner aspects of the ONL, where the rod nuclei are located [11, 13, 14]. However, to our knowledge, its impact on cones has never been investigated. We here show that the accumulation of immune cells was strongly associated with the loss of more than 50% of the cones. This loss was demonstrated by the disappearance of PNA^+^ cells (which might have suggested the loss of cone outer segments only) but also CAR^+^ cells, a marker that is found throughout the cone cytoplasm, demonstrating the loss of the cells. In accordance with the previously described anti-inflammatory properties of TSP1 [39–42], TSP1 severely reduced the numbers of accumulating infiltrating T cells, Mos, and Mφs, but interestingly not MCs, and very significantly reduced the mRNA level of cytokines at day 4, which strongly suggests they were expressed by the leukocytes. TSP1 inhibits retinal immune cell infiltration [40, 56, 57] and we previously showed that it accelerates macrophage elimination via its receptor CD47 [39]. Importantly, TSP1 treatment also significantly prevented the loss of cones at day 7 but failed to increase cone survival in cone-enriched cultures from chicken embryos, a model developed to characterize RdCVF [16, 32]. In these experiments, we used human TSP1, which is 85% identical with chicken TSP1, contains the same receptor binding sequences, and has been shown to equally inhibit chicken and human endothelial cell proliferation in vitro [43] at the concentration used in our experiments. The absence of a direct protective effect of TSP1 on cone survival strongly suggests that TSP1 protects cones in RD through the inhibition of immune cells, similarly to previous results showing inflammation-induced rod degeneration in RD [11, 13, 14].

We recently demonstrated that IL-1β, an inflammatory cytokine produced by MPs, induces rod-, but not cone-, death through the disruption of retinal glutamate homeostasis [58]. To our knowledge, the mechanism of inflammation-induced cone death is unknown. Cones are highly dependent on glucose uptake for survival [16, 17], which is regulated by RdCVF [16]. We here demonstrate that RdCVFL and RdCVF expression are dramatically reduced within 4 days of experimental RD, similar to human long-term detached retina [45]. In our experiments, the decrease in the expression of RdCVFL (expressed by rods and cones) and RdCVF (exclusively produced by rods [32]) is likely the result of transcriptional regulation and not due to rod loss, as the majority of rods are still present in 4-day RD. Interestingly, it affected both variants, RdCVF that interacts specifically with basigin 1 and promotes the transporter activity of GLUT1 [16] as well as RdCVFL, the long splice form, that contains the thioredoxin motive, and protects against oxidative stress [21, 22]. Both variants likely promote cone glucose uptake, as protection against oxidative stress has been shown to decrease the half-life of GLUT1 at the plasma membrane [24]. The RD-associated downregulation is likely in part due to the RD-induced inflammatory reaction, as Mos decrease RdCVF/RdCVFL expression in explants and inhibition of immune cell accumulation by TSP1 in RD significantly restores RdCVF/RdCVFL expression.

Glucose uptake in cones and their survival is not only regulated by RdCVF/RdCVFL, but also by insulin signaling [16, 17], evidenced by progressive cone loss in mice with cone-specific deletion of PI3K (p85, indispensable for insulin signaling) [25, 26]. Using insulin and a specific insulin receptor inhibitor on mouse retinal explants, we confirmed that insulin receptor signaling was essential for cone survival ex vivo.

In retinitis pigmentosa, where cones die secondary to the primary loss of rods and their RdCVF secretion, systemic administration of insulin improves glucose uptake in cones and inhibits their death, despite the absence of RdCVF [19, 20]. Similarly, we here show that insulin supplementation and the insulin sensitizer rosiglitazone, significantly prevented RD-induced cone loss, despite having no effect on the level of inflammation. In contrast, IGF-1 supplementation did not increase cone survival in RD, underlining the specificity of insulin signaling for this effect. Mechanistically, insulin receptor signaling increases the GLUT1-dependent glucose uptake via the activation of the mammalian target of rapamycin complex 1 (mTORC1) [19, 20]. In RD, glucose supply to cones likely becomes critical when RD-induced inflammation reduces RdCVFL and RdCVF expression and highly metabolic active immune cells compete for fuel in the photoreceptor cell layer [27]. Insulin supplementation might thereby increase glucose uptake to cones and prevent cone starvation in RD.

## Conclusion

In summary, our results describe a new mechanism by which inflammation induces cone death in RD. This mechanism might not be specific to RD but could also be involved in other retinal diseases characterized by chronic inflammation such as AMD [39, 46]. Therapeutic inhibition of inflammation and restoration of insulin signaling and glucose availability to cones might prevent cone malfunction and death in these diseases. Indeed, our study demonstrates that metformin, an insulin sensitizer with known anti-inflammatory effects [53, 54], significantly decreased inflammation and increased cone survival. Metformin or similar agents might prevent RD-associated cone death until the RD can be surgically repaired in the future.

## Data Availability

All data generated or analyzed during this study are included in this published article. The datasets used and/or analyzed during the current study are also available from the corresponding author on reasonable request.

## Abbreviations

RD: Retinal detachment
MP: Mononuclear phagocytes
RdCVF: Rod-derived cone viability factor
PI3K: Phosphoinositide 3-kinase
VMT: Vitreomacular traction
MH: Macular hole
PVR: Proliferative vitreoretinopathy
IL: Interleukin
IL-1ra: IL-1 receptor antagonist
CCL: Chemokine C-C motif ligand
CXCL: Chemokine C-X-C motif ligand
b-FGF: Basic fibroblast growth factor
G-CSF: Granulocyte colony-stimulating factor
GMCSF: Granulocyte/macrophage colony-stimulating factor
IFN-γ: Interferon-γ
MIP: Macrophage inflammatory protein-1
PDGF: Platelet-derived growth factor
RANTES: Normal T cell expressed and secreted
TNF: Tumor necrosis factor
VEGF: Vascular endothelial growth factor
TSP1: Thrombospondin-1
IGF-1: Insulin growth factor-1
DMEM: Dulbecco’s modified Eagle’s medium
HNMPA: Hydroxy-2-naphthalenylmethylphosphonic acid
DMSO: Dimethyl sulfoxide
PNA: Peanut agglutinin
CAR: Cone arrestin
IBA-1: Ionized calcium-binding adapter molecule-1
ONL: Outer nuclear layer
Th1: T helper type 1
MCs: Microglial cells
Mφs: Monocyte-derived macrophages
cMos: Classical monocytes
ncMos: Non-classical monocytes
